# Cerobro-Spinal Meningitis

**Published:** 1866-03

**Authors:** Geo. N. Duzan

**Affiliations:** Zionsville, Ind.


*Cerobro-Spinal Meningitis.* By Geo. N. Duzan, M. D., Zionsville, Ind.

This disease appeared in this vicinity first in the Spring of 1863, and it has not failed to appear annually since its first visitation. During the first year of its visitation it was regarded by a few physicians as "Congestive Remittent Fever." But those who so regarded it during the first year of its appearance were forced to change their opinion by the varied and unaccountable phases which the disease presented at the time of its second appearance, which was in the Spring of 1864. The appellation of "Spotted Fever" was then given to it by a few physicians, and by others more appropriately, because more expressive of its anatomical seat and pathological character, "Cerebro-Spinal Meningitis." The disease begins usually with a decided chill of varied duration and intensity, lasting from one to twelve hours, followed by febrile reaction, the grade of which is in inverse proportion to duration of chill. In many cases (about one of six) there is no reaction. In such cases the pulse is almost imperceptible from beginning of attack. A contracted state of the skin, cold, clammy perspiration, confined state of the bowels, frequently vomiting, coma and death. The absence of premonitory symptoms in such cases is a point of peculiar interest, and by resemblance in every particular, excepting confined state of bowels, to "Congestive Fever," it is frequently taken for that disease. But with the cold stage the resemblance to Congestive Fever terminates, and it is unmistakably distinct. The first evidence of reaction is an increase in volume and force of pulse, which is followed by a return of heat to surface of body, extreme jacittation, acute pain mostly referred to head and and particularly to occiput, frequently changing, however, from one point to another with incredible rapidity, increased sensibility of cutaneous surface, patient intolerant of slightest touch, rigidity of posterior cervical muscles with opisthotonos, eyes suffused, sometimes one pupil dilated, but generally both, with loss of vision; spots which appear upon both legs, arms and trunk are observed occasionally; no degree of pressure will alter their color; they are caused by extravasated blood, are not true eruptions, and they indicate malignancy of the disease; pulse but slightly accelerated, sometimes apparently undisturbed; bowels constipated; urine scanty, and contains an excess of phosphates. The disease will last from ten to forty days without any marked change in its phenomena. The phenomena which the disease presents are so numerous, and wanting uniformity, that every individual case may be properly regarded as a distinct disease. All of the phenomena, however, point unmistakably to the cerebro-spinal nervous apparatus as the seat of disease.

The disease is not contagious, because in selecting its victims it manifests a decided preference for young persons of an age from three to eighteen, and adults of a nervous temperament, and requires for its development a constitutional predisposition and susceptibility to the influence of an endemic poison. The nervous temperament and the several changes in organization as well as the natural mobility of the excitomotory system of childhood predisposes to nervous disorders, such as nervous pain and spasm, and are natural predispositions to Cerebro-Spinal Meningitis. Imperfect nutrition, confinement in impure air, continued exposure to cold, and retention of oxidized matter in the blood, will so change the constitution of blood that its cells will fail to carry an adequate supply of oxygen into the system, and the processes of calorification and histogeny are interrupted. A condition similar to venous hyperæmia ensues, the nervous centres are not supplied with vitalized blood, which is essential to healthy action, and they are thrown into a state of hyperæsthesia and excessive mobility which predisposes to endemic poisons. An individual having the natural and the acquired predisposition alluded to, if exposed to the depressing influence of malaria, the circulation in distant parts will be suspended, the extremities will become cold, whilst the head and internal organs will be congested. Coma and death, or (if the vital power be not too far exhausted,) inflammatory reaction will follow, and what parts are more likely to become the seat of the inflammatory action than the morbidly excited nervous centres ? If the theory of its etiology and pathology above expressed be true, the treatment is clearly indicated. During the cold stage, although stimulants are indicated, they are of but little benefit to patient. Owing to retarded circulation and congested state of internal organs, they either remain in the stomach entirely inert, or are ejected by vomiting. After reaction is established, such measures should be adopted as will equalize the distribution of blood and excitability. Counter-irritants to invite blood to extremities, and thus relieve internal organs, are of great utility. Opiates, owing to vascular excitement of nervous centres, invariably aggravate the pain. After an equilibrium of the circulation is established, and vascular excitement of the nervous centres removed, sulphate of quinia, stimulants and nutriment diet should be given.

These means of cure, varied as the nature of every individual case may require, constitute the treatment of the disease. It is, however, a fact both sad and humiliating, that notwithstanding all of our efforts almost one-half of those attacked die.—*Cincinnati Lancet & Observer.*

